# Spatio-Temporal Tolerance of Visuo-Tactile Illusions in Artificial Skin by Recurrent Neural Network with Spike-Timing-Dependent Plasticity

**DOI:** 10.1038/srep41056

**Published:** 2017-01-20

**Authors:** Alexandre Pitti, Ganna Pugach, Philippe Gaussier, Sotaro Shimada

**Affiliations:** 1 ETIS Laboratory, UMR CNRS 8051, University of Cergy-Pontoise, ENSEA, Cergy-Pontoise, France; 2 Energy and Metallurgy Department, Donetsk National Technical University, Krasnoarmeysk, Ukraine; 3 Dept. of Electronics and Bioinformatics, School of Science and Technology, Meiji University, Kawasaki, Japan

## Abstract

Perceptual illusions across multiple modalities, such as the rubber-hand illusion, show how dynamic the brain is at adapting its body image and at determining what is part of it (the self) and what is not (others). Several research studies showed that redundancy and contingency among sensory signals are essential for perception of the illusion and that a lag of 200–300 ms is the critical limit of the brain to represent one’s own body. In an experimental setup with an artificial skin, we replicate the visuo-tactile illusion within artificial neural networks. Our model is composed of an associative map and a recurrent map of spiking neurons that learn to predict the contingent activity across the visuo-tactile signals. Depending on the temporal delay incidentally added between the visuo-tactile signals or the spatial distance of two distinct stimuli, the two maps detect contingency differently. Spiking neurons organized into complex networks and synchrony detection at different temporal interval can well explain multisensory integration regarding self-body.

Accumulated evidence demonstrates the extraordinary lability of the body image, which is thought to emerge from the dynamic integration of signals from the different senses^1^,^2^,^3^,^4^. The redundancy of the signals and, in particular, their contingency is strongly believed to be captured for acquiring body image. However, its neural embedding should be robust enough to permit slightly incongruous signals to bind each other while retaining the ability to detect inconsistency for largely incongruous ones. For instance, *spatial* or *temporal* mismatches during visuo-tactile events can distort spatial judgment of the location of the body limbs, whereas its perfect congruence can enhance judgment of the spatial location^5^,^6^. Exemplary experiments are the rubber-hand illusion (visuo-tactile congruence), amputees with phantom-limb illusions, patients with spatial hemineglect, and asomatognosic patients who deny the ownership of their own limb (proprioceptive and efferent copy binding)^1^. These cases are based on malfunction of the detection of contingency in the ongoing sensorimotor information flow or in the virtual one reconstructed in the parieto-motor circuits. Perception of the contingency is accompanied by sense of agency and body ownership^7^,^8^,^9^, whereas its distortion can give rise to a sense of other, which is essential for inter-subjectivity. The congruence of multi-sensory signals has been acknowledged for self-perceptual experiences^6^. Among the studies that emphasize this aspect in the rubber-hand illusion (RHI), Shimada *et al*.^10^ showed that delayed visual feedback as short as 200–300 milliseconds can disrupt the illusion effect.

The perception of self-body in RHI is associated with different brain areas, mainly the parietal cortex interconnected to other regions like the premotor cortex or the extrastriate body area in the lateral occipitotemporal cortex as revealed by recent fMRI studies; e.g.^11^,^12^. Within the parietal cortex, neurons in the superior parietal lobe (SPL) and intra-parietal sulcus (IPS) are found more active during self-motion, whereas their activity is more attenuated as the delay lengthened^13^,^14^. In contrast, there is only a few neurons in these regions that show opposite responses to the delay and during the actions of others whereas they are found in a bigger proportion in the right inferior parietal lobe (rIPL)^13^,^14^. Based on these observations, many researchers have suggested a comparator model for self-assessment. In this view, a forward dynamic model predicts the consequences of motor commands, and these are compared with the actual feedback^15^. Depending on the degree of sensory discrepancy (e.g., the contingency between the afferent signals), one brain network will identify self-produced actions as our own actions or will recognize another person’s actions^16^,^17^.

However, the network identification cannot explain (*1*) why the peculiar 200–300 ms delays are detected or (*2*) the functional organization of the parietal cortex for self-other recognition that links the external biological motion (macroscopic level) with the internal neuron dynamics (microscopic scale). We propose to answer these questions with neurocomputational models. Because timing (i.e., synchrony, contingency, rhythmical patterns and temporal delays) is a crucial computational factor in neural networks, we believe that Hebbian learning is at the root of the temporal integration within sensorimotor networks^18^. More precisely, the biologically inspired mechanism of spike-timing-dependent plasticity (STDP^19^,^20^) can serve to detect the contingency at the neural level for categorizing the sensorimotor signals in a situation of interaction with others^21^. Because STDP permits reinforcement of the synaptic links between synchronous neurons and prediction of long sensorimotor patterns in real time^22^, the prediction level can serve for contingency detection and self-motion recognition^23^,^24^.

In this paper, we relate neural models with the previous work by Shimada *et al*.^10^ to elucidate how the rubber-hand illusion is performed in the parietal lobe and why it does not occur when the visual and tactile inputs are separated by 200–300 ms. Using an artificial skin, a video camera and recurrent neural networks of spiking neurons, we studied how multi-modal integration occurs within the networks and how spatio-temporal patterns are learned based on contingency detection. We assumed that the most congruent sensorimotor patterns strengthen their links more than the incongruent ones and that the activity level within the network is associated with the recognition of self or other. The comparison of the activity levels of these two clusters of neurons may enable differentiation of, to some extent, true synchronization from false ones. The main findings of our study is that the combination of STDP and recurrent NN can reproduce the limited attenuation of multisensory integration with temporally incongruent sensory inputs (150 ms) regarding the body, similarly seen in RHI. A recurrent multimodal network hierarchically organized into a complex network is more robust to delays and incongruous signals similarly seen in RHI than a simple associative map.

## Results

**Figure 1:**
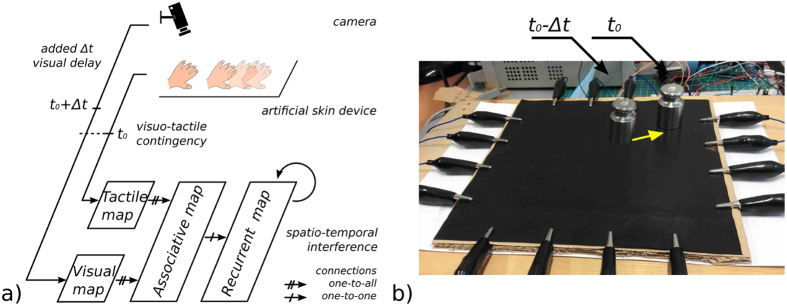
Experimental setup for modeling illusory effects in a visuo-tactile neural architecture of the parietal cortex using a tactile device and delayed visual feedback. In (**a**), the artificial neural networks receive the unimodal visual and tactile sensory inputs as incoming information. The first layer performs an initial pre-filtering, and the second layer consists of an associative map that binds the two unimodal neural populations. Finally, the third layer corresponds to a recurrent map that integrates over time the instantaneous visuo-tactile signals. This last network has the capabilities of a working memory to resist noise and temporal delays, as it governs the internal representation of the parietal cortex. In (**b**), visual temporal delays on the camera are equivalent to the spatial tactile displacement on the artificial skin.

The learning process within the neural maps is performed sequentially by presenting instances of visuo-tactile signals when the hand (contact point of 2 *cm*^2^) moves above the tactile device and is always in contact with it, under the vision field of the camera (see Fig. 1 and the Device and Methods section).

### Receptive Fields of Associative and Recurrent Maps

**Figure 2:**
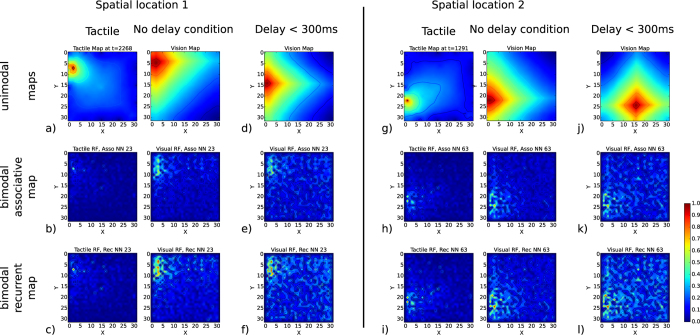
Receptive fields for two different spatial locations of the contact point in the unimodal and bimodal tactile and visual maps. The RFs for the asso. and rec. maps are displayed during the no-delay and delay conditions (300 *ms*). (**a**) and (**g**) correspond to the strict visual and tactile spatial RFs of the unisensory maps taken at one snapshot for two arbitrarily chosen locations. (**b**) and (**h**) correspond to the spatial RFs of two bimodal neurons of the asso. map firing the most for the visuo-tactile locations in (**a**) and (**g**). (**c**) and (**i**) correspond to the spatial RFs of two bimodal neurons of the recurrent map firing the most for the visuo-tactile locations in (**a**) and (**g**). Plots (**d**–**f**) and (**j**–**l**) are the neurons’ activity in the asso. and rec. neurons, when a visual delay of 300 *ms* is added.

Each map learns its proper incoming signals so that we can observe functional differences between the unimodal maps, the associative (asso.) and recurrent (rec.) maps to represent the visual and tactile as well as the visuo-tactile receptive fields (RFs), see Fig. 2. To better understand how the neural maps behave for different initial conditions, we plotted the neural map activity for two different spatial locations of the contact point, which means there were two different RFs. The first two plots in Fig. 2(a) (resp. g) display the tactile and visual RFs associated with the two unimodal maps recorded for one particular location. The next two plots in Fig. 2(b) (resp. h) present the visuo-tactile RFs associated with the activity of one selected bimodal neuron in the asso. map, which is also the most active for that particular location. The two plots presented in Fig. 2(c) (resp. i) correspond to the spatial RFs of one selected bimodal neuron in the rec. map, which also corresponds most closely to that particular spatial location. The respective plots in Fig. 2(e) and (f) (resp. k and l) are the corresponding visual RFs of the asso. neuron and of the rec. neuron when a visual delay of 300 *ms* is added; see plots in (d) and (j). These sets of graphs show that the asso. and rec. neurons have similar visual and tactile RFs located in the upper-left area. The asso. map successfully learns the bimodal correspondence between the spatial RFs of each unimodal map as well as their spatial limits; for instance, the bimodal association is restricted to a small tactile area and to a small visual area, in comparison with the unimodal RFs. At the same time, we observe that the RFs of the asso. neuron are slightly different from their current location in the unimodal map, whereas the RFs of the rec. neuron cover a broader area, similar with the spatial range of the stimulus in the unimodal maps. Moreover, when a visual delay of 300 *ms* is added, the two maps show some added noise in the spatial estimation of their respective RFs.

We propose that at some points the bimodal maps show how the asso. and rec. maps have learned a unified multimodal representation. These differences between the two maps can reflect some functional differences in the detection of visuo-tactile contingency and in the processing of erroneous signals or illusory events. The comparison between the two maps or their dynamic registration can serve then to better detect the contingent signals.

### Visuo-Tactile Interference Patterns of the Neural Activity

**Figure 3:**
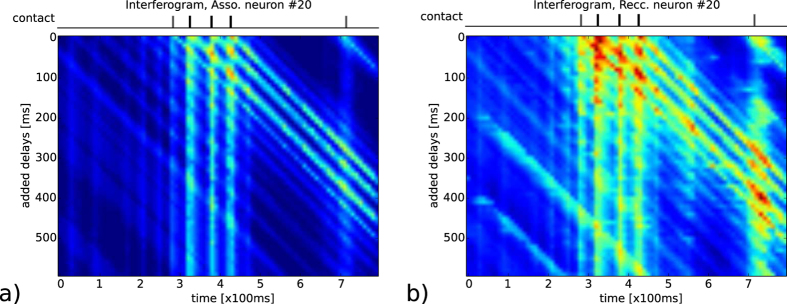
Interference patterns of the neural activity in the associative and recurrent maps for various visual delays of up to 600 *ms*. In (**a**), the associative neuron’s activity is weakly sensitive to the interference patterns due to contiguous visuo-tactile activity between the vertical lines (current tactile input) and the diagonals (visual delays). In (**b**), the recurrent neuron is more sensitive to the visuo-tactile contingency as the activity level increases, although it also discharges in advance or later when strict contingency is not respected (memory effect).

The plots in Fig. 3 show a novel method that we propose and name an interferogram. As an analogy with signal processing, we propose to study the *interference patterns* that one signal makes on another when they are combined with respect to delays; how delayed visual signals interfere with tactile signals in the bimodal neurons of the asso and rec maps? The analysis of interferograms can help us better understand how delays influence the occurrence of a signal degradations (negative interferences) and signal enhancement during illusion effect) at the neuron level and for delays in the interval range between [0, 600 *ms*]. The interferograms in Fig. 3 present the dynamics of two neurons plotted for visual delays up to 600 *ms* (vertical axis) from the associative map in a) and from the recurrent map in b).

A strong vertical activity of the neuron indicates the spatial proximity of the visuo-tactile stimulus to the neuron receptive field, whereas a lower vertical activity of the neuron indicates the situations of distal and non-congruent tactile stimuli. Therefore, the vertical lines represent the sensitivity of the neuron to the immediate tactile stimulation (its tactile receptive field), whereas the diagonal lines represent the sensitivity of the neuron to the delayed visual input (its visual receptive field). Strong activity in the vertical lines indicates that the network holds visual input for a while so that immediate tactile stimulation can fire the neuron, whereas strong activity in the diagonal lines indicates that the network holds tactile input for a while so that delayed visual input can fire the neuron. This property is more prominent in rec. neurons than in asso. neurons, where the former behave more like a working memory for the self-body image, with anticipatory and hysteresis effects. The working memory effect is due to the recurrent links in the rec. map, which permit the neurons to learn spatio-temporal sequences, although the temporal interval of each neural pair is limited to only 50 *ms*, as fixed in the experiment (see Devices and methods).

**Figure 4:**
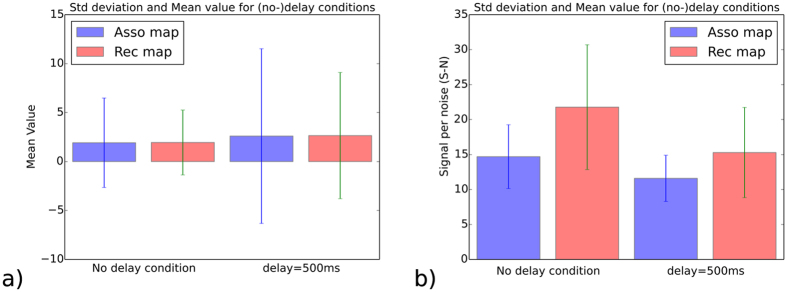
Mean and the standard deviation for the asso. and rec. maps and their confidence level (the signal to the noise), resp. (**a**) and (**b**). (**a**) The graph presents the mean and variance of the neural activity in the non-delay condition (on the left side) and in the situation of a visual delay of 500 *ms* (on the right side). Visual delays influence the amplitude discrepancy of this measure for the two maps and increase the variance, twice more for the asso map than for the rec map. (**b**) This measurement quantifies the confidence level of the neural maps to the input stimuli with respect to non-delayed and delayed visual feedback (500 *ms*). It is computed as the maximum activity minus the local field activity. This measure shows that at the population level it is possible to detect contingency by comparing the activity to a threshold value.

At the neural mass level, the mean and the standard deviation of the neurons for each map can provide a metric of the confidence level of the neurons for contingency detection; see resp. Fig. 4(a,b). In Fig. 4a), the variations between the situation in the no-delay condition and the situation in the delayed condition (500 *ms* visual delays) show that the variance is increased in the case of the delay condition, almost the double. Moreover, the recurrent map is more robust to delays than the associative map as it has a lower variance (one-third lower), the confidence level is therefore better in this case. This suggests that the comparison of the neural activity to a threshold value can permit detecting temporal discrepancy and determining with a confidence level whether an illusion has occurred.

The confidence level computed in Fig. 4b) corresponds to the mean and the standard deviation of the difference between the most active neurons with respect to the local field potential. It can provide a metric of the signal to the noise at the population level for contingency detection. This graph shows similar results to Fig. 4a) for which, the variations between the situation in the no-delay condition and the situation in the delayed condition for the confidence level is diminishing in the case of the delay condition, with a higher value for the rec map.

**Figure 5:**
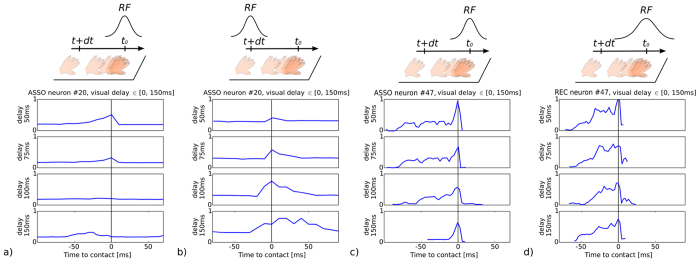
Visual delay sensitivity of one neuron in the associative map in conditions of spatially non-contiguous and contiguous visuo-tactile signals; (**a**) and (**b**), respectively. Comparison between visual delay sensitivity of one neuron in the associative map and in the recurrent map; (**c**) and (**d**). Adding a temporal delay gives different neural activities whether or not the visuo-tactile receptive fields coincide. In the case of a spatial mismatch of the visuo-tactile receptive fields as in (**a**), the neuron activity decreases, cancelling the perception of contingency. In the case of a spatial overlap of the visuo-tactile receptive fields as in (**b**), the neuron activity increases, giving the illusion of contingency. Besides, the recurrent neuron in (**d**) is more robust to delays than the asso neurons in (**c**).

We further investigated this issue with the use of temporal delays in the visual input to manipulate the visuo-tactile associations within the networks. Any temporal delays between the visual and tactile signals will distort the activity level within the neural maps, although the amplitude level cannot provide clear insight into the presence or absence of an illusion. As an example, the plot in Fig. 5(a,b) shows the neural activity of one neuron in the associative map selective to one spatial location when the hand is entering its area of influence or leaving it and for various visual delays up to 150 *ms* whereas the plot in Fig. 5(c,d) shows the neural activity of one neuron in the associative map and of one neuron in the recurrent map when the hand is entering their area of influence and for various visual delays up to 150 *ms*. The vertical black line corresponds to the time-to-contact, which is the period when we enter in the tactile neuron’s receptive field. The two plots (a-b) correspond to the situation when the spatial location of the hand with the visual feedback delay is outside from the spatial location of the neuron’s visual RF in Fig. 5(a) and when the spatial location of the hand with the visual feedback delay is inside the spatial location of the neuron’s visual RF in Fig. 5(b). The plots Fig. 5(c) and (d) is a comparison of the behavior between the asso neuron and the rec neuron, which shows an anticipatory effect and a much stronger activity of the rec neuron before the time-to-contact than the asso neuron.

Although a visual delay has been added, the graphs in Fig. 5(a,d) show that the neuron always fires when the hand is moving within the neuron’s tactile RF; i.e., the time-to-contact on this spatial location in the tactile sheet. Nonetheless, the behavior is different for the two maps. For the asso map, the signal degradation is caused by the spatial distance between the current location of the hand (tactile response) and the visual location in the delayed image (visual response). By contrast, for the recurrent map, the signal enhancement is due by the network property of the recurrent map (its recurrent links), which has bigger receptive fields. These latter situations Fig. 5(b) and (d) may correspond to an illusory effect: the spatial proximity of each RF enhances the neural activity, giving the illusion of temporal contingency, although a temporal delay was added.

On the one hand, when the visual and tactile RFs are misaligned, which corresponds to Fig. 5(a), gradually adding a delay greater than 50 *ms* has the direct effect of diminishing the neural activity level, although the precise timing of the time-to-contact of the hand entering the neuron RF is preserved. On the other hand, in situations of spatially contiguous visuo-tactile RFs, which correspond to Fig. 5(b), we can observe the counter-intuitive result of an *increasing* neural activity when visual delays are added, even though the precise timing of the time-to-contact is preserved. In this situation, the spatial congruity gives the illusion of temporal contingency.

### Neural Property with Respect to Visual Feedback Delay

**Figure 6:**
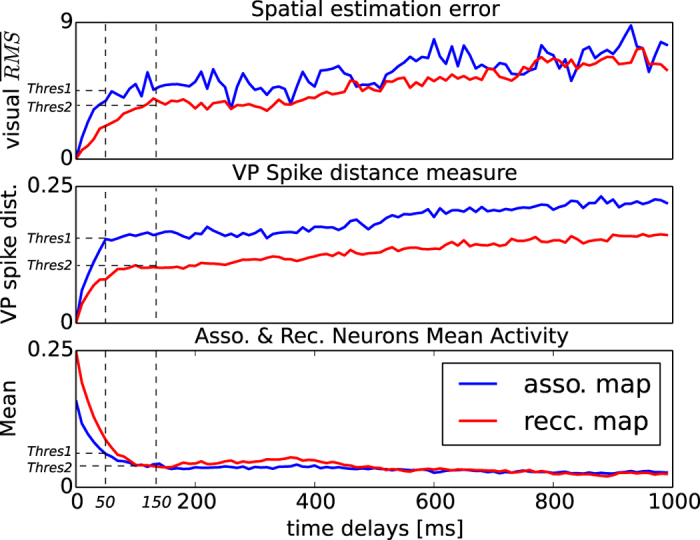
Average neural activity of the associative and recurrent maps with respect to visual delays and for spatially congruent visuo-tactile signals (bottom), temporal spike precision relative to visual delays added with the VP distance (middle), and visual spatial error with respect to delays (top). For the three plots, the activity level quadratically decreases for visual delays less than 150 *ms*. This interval corresponds to the sensitivity of the neurons within their RF, for which the rec. map is more robust than the asso. map to cancel out the effect of delays with lower spatio-temporal error and with less variability (middle and top charts). The asso. map, which is more sensitive than the rec. map, has an amplitude level less than 50 *ms*. With a delay longer than 150 *ms*, the neurons of the two maps linearly decay.

To analyze the statistical properties of the two maps, we measure how their neural dynamics behave with respect to the visual feedback delays. The plot in Fig. 6 shows the spatial estimation in the asso. and rec. maps, their synchronization level, and their amplitude level with respect to delays; the top, middle and bottom charts, respectively. These measures of the delayed neural activity were analyzed and compared with the neural activity retrieved when the hand is inside each neuron’s tactile RFs at the time-to-contact in the zero-delay condition, which occurred when the neuronal signals are above the threshold value of 1.5, as heuristically chosen. The neuron spikes above this threshold correspond to the vertical lines displayed in Fig. 3(a,b), when the current visuo-tactile signals are within its receptive field. The bottom chart presents the congruent mean activity for the recurrent map (resp. associative) as a red line (resp. in blue line) for visual delays less than 1000 *ms*. The middle chart displays the spike distance measure proposed by Victor and Purpura (VP), which computes the spike variability and the level of phase synchronization between two spike trains with a cost function^25^. The VP distance was calculated between a spike train in the non-delay condition and its corresponding spike train in the delayed condition. The top chart corresponds to the visual spatial error estimated for all the neurons with respect to delays and calculated as the euclidean distance between the visual spatial position estimated during the non-delay condition and the current visual spatial position retrieved during the delayed condition.

The graphs present three different neural regimes with respect to the visual delay added. Below 50 *ms*. the first regime can be defined with a power-law function with a rapid discrepancy in the neurons’ dynamics for the asso. map only. Below 150 *ms*., the second regime can also be defined with a power-law function but with a slower discrepancy, for the rec. map only. Above 150 *ms*, however, the two curves confound each other or present similar trends for the three charts. These three different neural regimes characterize the conditions that determine whether the illusory effects occur.

During the discrepancy stage for delays <150 *ms*, the neural signals are inversely proportional to the visual delay added; therefore, adding a visual delay affects the gain level of the neurons, which means that the two neural maps are sensitive to visual delays. The two maps have similar trends in the bottom chart but different amplitude levels. These differences can also be seen in the two other charts, where the VP distance indicates a better response of the rec. map than the asso. map to phase-synchronize to the correct signals even in the presence of delays. The top chart shows a spatial estimation error or a spatial drift of the visual location of the target relative to delays. The RFs of the neurons in the rec. map are sensitive to a larger spatial area than those of the asso. map. In the temporal domain, the response characteristics of the rec map to visual delays in the bottom graph are slightly higher than the asso map during the 200–500 ms interval although no t-tests were performed. In comparison to the associative neurons, the robustness of the rec. map can be explained by its recurrent links, which form neural groups capable of sustaining longer spatio-temporal sequences chained dynamically, better anticipating the spatio-temporal memory traces and recovering from erroneous signals.

These three results adequately support the observations made on the asso. and rec. maps for multimodal integration and spatio-temporal binding in RHI-like experiments; i.e., the decrease in neural activity, phase lags and spatial estimation errors. The comparison of the two threshold values of 50 *ms* and 150 *ms* for each parameter (spatial, temporal and amplitude-level) and for the two maps may make it possible to distinguish the first case of self-recognition during the illusion (both thresholds below 50 *ms*) from the second case of illusion perception and its detection at the same time (for the intervals 50 *ms* and 150 *ms*).

Above 150 *ms*, the neural signals present low but stable dynamics (bottom chart). A similar trend is depicted in the two other charts, with a static phase lag and visual error, which correspond to the temporal limit of the illusory effect. The stable VP distance corresponds to phasic errors for the two maps, and if the rec. map has a lower VP index, it predicts more accurately than the asso. map. These temporal errors have some influence in the spatial estimation (top chart), with more fluctuation for the asso. map. The two maps have similar trends for the three measures, with variations proportional to the delays. This interval range is above the limit of the contingency perception of the delayed signal.

### Small-world Network Property in Recurrent Map

**Figure 7:**
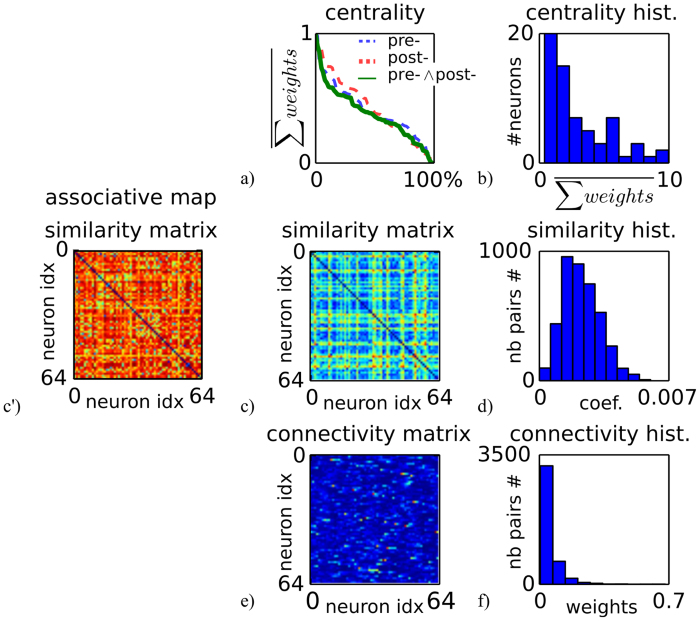
Measures and indices to characterize the functional organization of the recurrent neural network based on complex networks and graph theory. (**a**,**b**) The centrality index describes the relative effectiveness of neurons within the network in terms of the number of pre-synaptic and post-synaptic connections. The power-law curve is characteristic of small-world-networks, where the most connected neurons represent the network’s “hubs.” (**c**-c’-**d**) The similarity index measures the similarity among the neurons by comparing their weights; asso map in (c’) and rec map in (**c**). An important mass of neurons constitutes the network, which guaranties some redundancy, but the less similar ones potentially correspond to the most critical ones. (**e**,**f**) The connectivity matrix permits identification of the unidirectional connections or causal links between the pairs of neurons. This method is complementary to the centrality measure. The stronger the value of this index, the stronger the influence of the neuron on its associated neurons. Again, the logarithmic curve of the histogram is characteristic of complex and hierarchical networks.

To understand better how the functional organization of the rec. network and its topology relate to each other, we analyzed three quantitative methods taken from graph theory and complex networks^26^. Figure 7 shows different indices that characterize the topology of complex networks in general and of the neural circuits found in the human brain in particular^27^,^28^,^29^. These measures are named the centrality index, the similarity index and the connectivity index for the neurons of the rec. map; Fig. 7(a,b), (c-c’-d) and (e,f), respectively. We want to explain why the recurrent map is more robust against small delays than the associative map as showed in Fig. 6. It is important in order to characterize multimodal networks based on timing in associative areas such as the parietal cortex. The measures presented show that the recurrent network splits the neural population into two class of neurons, neurons with strong bimodal coupling and neurons with loose bimodal coupling. This topology corresponds to a complex network demonstrating nonlinear behaviors as in perceptual illusion.

The centrality index plotted in Fig. 7(a) defines the density distribution of neurons within the network that are found central to it. These neurons are the most connected ones (the green line), receiving the most information from the upstream signals of the pre-synaptic neurons and propagating the most to the downstream signals of the post-synaptic neurons (the blue and red dashed lines, respectively). The centrality index indicates that few neurons within the network (less than 10%) are highly connected and possess a high score, whereas the majority of the neurons have an average or a very low score. These latter neurons are at the periphery of the network in comparison to the most connected neurons, which are few in number. The logarithmic curve in the histogram plot in Fig. 7(b) is typical of small-world networks^28^,^29^.

The similarity index in Fig. 7(c-c’) is defined as the inverse of the distance between the neurons’ weights computed for all neuron pairs. In complement to the similarity index of the rec map in Fig. 7(c), we add the similarity index of the asso map in Fig. 7(c’) for comparison. The matrix for the asso map shows that there exists a lot of redundancy among the neurons that overly encode bimodal signals, which are then difficult to separate or discriminate. This topology is different from a small-world network. The rec matrix in (c) instead is sparse, which is characteristic of a hierarchical organization within the network, with the neurons as part of isolated groups. The histogram in Fig. 7(d) shows that a large population of neurons are part of the same group, as they have more or less the same similarity index centered around the value 0.002, with some neurons very similar to each other (similarity index above 0.004) and others very unique (similarity index near 0.00). As for the centrality measure, these latter two groups differ from the bulk of the neuron, and can therefore correspond to distinct functional behaviors within the network.

Finally, the connectivity matrix plotted in Fig. 7(e) is defined as the one-to-one and unidirectional connection strength between two neurons taken from their synaptic links. In comparison to the two previous qualitative measures, the connectivity index is another measure of the importance of particular neurons at the network level. In accordance with them, this measure again informs about the importance of some neurons, with a histogram of the connectivity index plotted in Fig. 7(f) following a power-law curve as for the centrality index, typical of a small-world network.

## Discussion

Our current experiment attempts to replicate visuo-tactile illusions such as the so-called RHI experiment to understand how neurons establish a unified representation by means of visuo-tactile experiences and how delays can extinguish its perception. Although it is not explicitly labeled as “self-body”, and that the neural activation does not have a subjective feeling of RHI reported in the literature^30^, the visual hand that coincides with tactile sensation should be understood as a unified perception, and our neural nets learned this task properly. Our main message is that STDP and a recurrent network can reproduce the attenuation of multisensory integration with temporally incongruent sensory inputs (150 ms) regarding the body and that spiking neurons organized into complex networks can duplicate timely-based as well as distorted signals similarly seen in RHI. We summarize in Table 1 the different situations for attenuation of multisensory integration depending on the visuo-tactile delays on the two maps, which may correspond to self recognition for real or perceived illusions or as other when no illusions are perceived.

The same mechanism for detect (in)congruencies in predicted and actual sensory action feedback may serve for self-other distinction. Each neuron learns its own visuo-tactile RF, which permits detection of the contingency of the visuo-tactile signals so that the temporal delays and spatial distance to its respective RF can affect the amplitude level of the neurons per se. The amplitude level of the neuron describes the conditions for self-body detection for inducing perceptual illusions such as in the RHI. Considering the difference between the two maps, the recurrent neurons are very robust to delays, sometimes with a tolerance of 150 *ms* or so, whereas asso. neurons sometimes showed fluctuations in activity with a delay shorter than 50 *ms*. Even the interval within 200–500 ms is slightly differentiated between the amplitude values of the rec map and of the asso map in Fig. 6 in the bottom chart. This information may serve to sketch a conceptual model regarding the neural mechanisms involved in spatial estimation of multimodal events as during RHI or even ventriloquism.

### Neurons anchored in the tactile receptive fields

Our experimental results show that visuo-tactile neurons are anchored in tactile receptive fields as can be inferred from the interferograms in Fig. 3, where vertical lines indicate the neuron sensitivity to tactile RF. The diagonal lines instead indicate the neurons’ sensitivity to only the visual RF. The rec. map is noticeably more tolerant than the asso. map to contradictory locations of the RFs and is therefore more robust to visual delays, as can also be seen from the better neural responses of the rec map in Fig. 3 with respect to the visual delays. We can understand that the functional role of those neurons is to translate a spatial distance from their visuo-tactile RFs into an amplitude variation, and temporal delays can also modulate their responses. Therefore, a “spatial distance” between the visuo-tactile signals or a “time lag” between the two modalities can be seen as variables that can be equally interchanged. Nonetheless, their relationship is non-linear, so within the limit of 150 *ms*, the neural amplitude level can convey information about the distance of one object to the RF. This distance measure can serve for, for instance, perceiving the body in its own reference frame and the space around it; the so-called peri-personal space is important for body ownership as well as for reaching objects nearby and defensive behaviors for object avoidance^31^.

The learning of visuo-tactile integration is rapid in the neural networks, which is in line with previous observations and models in favor of an acquisition at an early stage of somatotopic and visuo-tactile body maps^32^,^33^,^34^,^35^,^36^. Experiments with infants as old as 6 months show their sensitivity to small temporal delays for self-body registration and for self-other differentiation^7^,^8^.

### STDP and contingency detection

The temporal coherency needed for the neuron to be fired is different for the simple associative map and for the recurrent map, which also describes a difference in their functional organization.

**Figure 8:**
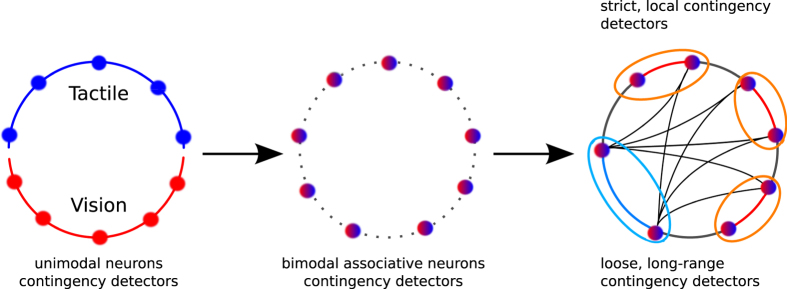
The integration of visual and tactile signals in the associative map and then in the recurrent map permits to be robust to spatio-temporal disturbances of distal multimodal events. The organization of the recurrent map into a small-world network can explain the synaptic multisensory integration for loose contingency detection; graphics inspired by^28^,^38^.

In our experiments, the amplitude level of the neurons characterizes the visuo-tactile contingency level, which is in line with the results found in fMRI studies showing the existence of contingency detectors for self-motion in experiments similar to those with the delayed RHI^10^,^16^. At the brain level, the mechanism for contingency detection in multisensory neurons is often attributed to the neural mechanism of spike-timing-dependent plasticity^37^ (STDP) because the precise timing of a pre-synaptic neuron can determine whether a post-synaptic neuron is fired. With respect to sensorimotor networks, we propose that STDP supports the release of contingency detectors at the millisecond order so that the temporal coordination of groups of neurons can describe a certain level of self-motion prediction, which can be used at the population level for error prediction; e.g., for self-assessment of body motion^37^ or limb ownership^24^. The organization of the recurrent network follows a small-world network structure so that few neurons can integrate and anticipate slightly distant multimodal events (loose contigency detection) with respect to the majority of neurons that can encode only unique multimodal events (strict contingency detection), see Fig. 8.

### 150 ms contingency discrepancy and the peri-personal space

One critical result is the 150 ms contingency discrepancy found in the visuo-tactile neural networks, which is very near the temporal constant of 100–140 ms found in the recording of event-related potentials during tactile remapping experiments^38^,^39^ and the temporal responses found by Shokur and colleagues to virtual touches of neurons in S1 and M1 during RHI, which occurred 50 to 70 ms later than those to physical touch, whereas V-only responses occurred 90 ms after the stimulus^40^. These intervals are also similar to the 200–300 ms found in the delayed RHI task in a previous study^10^.

The 150 *ms* temporal discrepancy can be understood as the limit of the visuo-tactile integration of the neurons’ respective RFs; see Table 1 *cases* #1 and #2. This temporal limit represents the visual spatial error with respect to the tactile RF, which corresponds to its area of influence at this location when an object enters this region^41^,^42^. Above this limit, any visuo-tactile signal is considered outside its area of influence and the multimodal integration effect is not perceived; see Table 1 *case* #3.

This result agrees with the idea that reference frames and anatomical and external spatial coding are concurrently active or interfering, and the dominance of one reference frame over the other and the integration of different reference frames are based on sensorimotor contingencies^38^. However, this work does not address the problem of coordinate transformations between different modalities in multisensory integration. Previous works done by the authors model the mechanism of gain-modulation found in parietal neurons for audio-visual and visuomotor coordinate transformations^43^,^44^. In future works, one attempt will be to extend this model to coordinate tranformation of visuo-tactile and proprioceptive reference frames for simulating RHI with a robotic hand.

### The comparator/identification model

Our architecture relies on the functional organization of two different maps–the asso. map and the rec. map–which provide different types of information that can be combined with each other; see Table 1. We propose that parietal neurons can use these mechanisms during self-motion as well as during other-motion^10^,^45^.

For instance, we may compare the amplitude level between these two maps to falsify self-based motion from someone else’s motion. Below 50 ms, the contingency detection is strict and is validated two times by the two maps; see Table 1 *case* #1. In the interval 50 ms–150 ms, the contingency detection is weaker and validated only one time by the rec map; see Table 1 *case* #2. A comparator model would require at least 150 ms to wait for the end of the process for the two maps. However, a more elaborate version of it, an *identification layer*, would require even more time if it had to identify (*1*) the nature of the input signals received and (*2*) how far they are from the expected ones^15^. This idea is an extension of the comparator model proposed by Blanke and others^9^,^46^, as well as Hiraki and colleagues, for body ownership^8^,^14^, in which the parietal cortex is at the-forefront of comparing and distinguishing the even of integrated proprioceptive/multimodal information from the odds of non-strict contingent information, see Fig. 8. The rIPL may be a candidate for detecting (in)congruencies in predicted and actual sensory action feedback and its structure may be organized as a small-world network (right figure).

On the one hand, the contingency detection performed by the asso. map (middle) is rather strict because a small temporal perturbation can affect the neurons’ integrity. On the other hand, the contingency detection performed by the rec. map is robust to account for larger temporal perturbations.

## Devices and Methods

### Experimental Setup

Our experimental setup replicated the settings of the rubber-hand illusion experiment^10^,^47^ and employed the apparatus we used in ref. 23, with neural networks receive the incoming signals from a haptic device and a video camera, see Fig. 1(b).

Our setup consisted of a conductive tactile device with a camera mounted on it that captures the contingent visuo-tactile signals from the experimenter’s hand moving above the artificial tactile sheet and continuously touching it on one point with a metallic weight, see Fig. 1(b). The experimenter was free to move his hand in all directions at a variable speed for a period of 5 minutes and at a system sampling rate of 30 *Hz*. **New** This sampling rate is low due to the signal processing done by the device but it is enough for detecting tactile displacement below the centimeter by the neural network. The raw incoming signals are sent to the neural networks, which then attempt to combine the visuo-tactile signals; see Fig. 1(a).

### Tactile Device

The haptic device consists of a pressure-sensitive conductive sheet with 16 electrodes placed on its boundary, see Fig. 1(b). Its implementation is explained in refs 48, 49, 50. The voltage of the electrical current injected into each pair of electrodes is read out, and the potential distribution on the global surface of the sensor sheet is estimated based on the inverse analysis of the local resistance in each pair, called electrical impedance tomography (EIT). Using this method, it is possible to detect any change in the resistance distribution of the material and to identify locations where pressure is being applied on the sensor sheet or to determine when it is stretched.

The sensor has a reasonable sensitivity threshold and can detect forces greater than 1 *N*. Hence, it can also detect tactile stimuli larger than 1% of the sensor area, which was an acceptable resolution for our experiment. The frame rate of the device is 10 *ms*.

The camera resolution is 320 × 240 pixels and the pixels’ colors are converted into gray intensities. The camera is fixed in front of the tactile device in the same position of the subject’s eye field in the RH experiment so that the spatial coordinates in the visual eye field and in the tactile sensor sheet correlate with each other. Its frame rate is set synchronized to the tactile device, which is 10 *ms*.

### Neuron Definition and STDP-like Algorithm

In the four neural networks we used a variant of the Hebbian algorithm, the rank order coding algorithm, which effectively grasps the structure of the spike-timing-dependent plasticity algorithm and of the classical Delta rule in the spatio-temporal domain^51^.

STDP has been found to modulate the neural activity of temporally related neurons in many brain regions by reinforcing their links. The rank order coding (ROC) algorithm was proposed by Thorpe and colleagues as a discrete and faster model of the derivative integrate-and-fire neuron and of the standard STDP reinforcement learning algorithm^52^. The rationale is that ROC neurons are sensitive to the sequential order of the incoming signals, that is, their *rank code*. The distance similarity to this code is transformed into an amplitude value. The scalar product between the input’s rank code and the synaptic weights then furnishes a distance measure and the activity level of the neuron. More precisely, the ordinal rank code can be obtained by sorting the signals’ vector relative to their amplitude levels or to their temporal order in a sequence. If the rank code of the input signal perfectly matches that of the synaptic weights, then the neuron fully integrates this activity over time and fires. By contrast, if the rank coding of the signal vector does not properly match the ordinal sequence of the synaptic weights, then the integration is weak and the neuron discharges proportionally to it. However, the ROC algorithm modulates the activity of one neuron with respect to the proper order or phase of its afferent sensory signals; in a sense, the rank code preserves the signal’s information structure (i.e., its phase). In this respect, this mechanism captures the intrinsic property of cortical neurons.

The neurons’ output *v* is computed by multiplying the rank order of the sensory signal vector *I, rank(I*), by the synaptic weights *w*; *w* ∈ [0, 1]. For a vector signal of dimension *M* and for a population of *N* neurons (*M* afferent synapses), we have


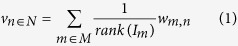


The updating rule of the neurons’ weights is similar to the winner-takes-all learning algorithm of Kohonen’s self-organizing maps^53^. For the best neuron *win* and for all afferent signals *m* ∈ *M*, we have


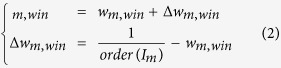


### Neural architecture

The neural architecture consists of four maps arranged as in Fig. 1(a). In the first stage, the unisensory maps learn to categorize their respective inputs into unimodal receptive fields (32 × 32 neurons each). In the second stage, an associative map (asso. map) learns the instantaneous coupling between the neurons of the two unisensory maps (64 neurons). Finally, in the third stage, a recurrent map (rec. map) encodes a temporal sequence from the associative network. The temporal horizon for each synaptic link is less than 50 *ms*, which is therefore the maximum possible time length of the synaptic links. This parameter is important in the simulation. The rationale is that the learning interval of STDP and the average synaptic length in the cortical maps are less than 50 *ms*^20^. This last map then models the properties of a working memory as it could be performed in the superior parietal cortex (rec. map)^54^ (64 neurons).

The EIT tactile matrix is directly fed into the neural network as the tactile input. The number of neurons in the tactile network is a 32 × 32 matrix, and it is chosen to be lower than the number of tactile elements so that the whole network can learn to generalize the entire mesh.

In parallel, the visual map receives pre-processed signals from a camera device to detect motion within the image. The visual network is of the dimensions 32 × 32, as is the tactile map.

The associative layer, which receives information from the two previous maps, is downsized to a network of only 64 neurons. The recurrent map also possesses 64 neurons, except that it receives as input the temporal buffer of its own activity over a period of time of 50 *ms*, which corresponds to a [64 × 5] input vector (10 *ms* sampling time). The neurons of the associative map are connected to the neurons of the recurrent map by directly adding their current dynamics to the values of the output neurons of same index multiplied by 0.5; 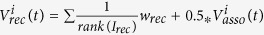.

### VP spike distance

Victor and Purpura proposed a measure of spike-train synchrony by computing the minimal cost necessary to transform one spike train into another by means of basic operations (spike deletion, spike insertion, spike shift)^25^. Each basic operation costs 1, which makes the distance sensitive to the timing of the individual spikes (phase synchronization).

## Additional Information

**How to cite this article**: Pitti, A. *et al*. Spatio-Temporal Tolerance of Visuo-Tactile Illusions in Artificial Skin by Recurrent Neural Network with Spike-Timing-Dependent Plasticity. *Sci. Rep.* **7**, 41056; doi: 10.1038/srep41056 (2017).

**Publisher's note:** Springer Nature remains neutral with regard to jurisdictional claims in published maps and institutional affiliations.

## Figures and Tables

**Table 1:** Summary of the different situations found for the asso. map and the rec. map depending on their neural activity and their relationship to temporal delay thresholds, their perceptual experiences and the brain areas associated with them.

	Asso map Threshold 1	Rec map Threshold 2	Perceptual Experience	Illusion
*case #1*	≤50 ms	≤150 ms	Self	Perceived
*case #2*	>50 ms	≤150 ms	Self-Other	can be perceived
*case #3*	>50 ms	>150 ms	Other	Not perceived

Depending on the neural activity within the two maps, two thresholds–below 50 ms and below 150 ms–are found for the asso. map and the rec. map, respectively. In case #1, below 50 ms for the two maps, the multimodal event, illusory or not, is perceived. This experience may bes associated with RHI. In case #3 above 150 ms, the multimodal event is not perceived. In case #2 in between, only the rec map is active, and the ambiguous signal (illusion) can be perceived and may be detected as well as fake.
